# Mitochondrial dysfunction in cell senescence and aging

**DOI:** 10.1172/JCI158447

**Published:** 2022-07-01

**Authors:** Satomi Miwa, Sonu Kashyap, Eduardo Chini, Thomas von Zglinicki

**Affiliations:** 1 Newcastle University Biosciences Institute, Ageing Biology Laboratories, Newcastle upon Tyne, United Kingdom.; 2 Department of Anesthesiology and Perioperative Medicine, Mayo Clinic, Jacksonville, Florida, USA.; 3 Signal Transduction and Molecular Nutrition Laboratory, Kogod Aging Center, Department of Anesthesiology and Perioperative Medicine, Mayo Clinic College of Medicine, Rochester, Minnesota, USA.

## Abstract

Mitochondrial dysfunction and cell senescence are hallmarks of aging and are closely interconnected. Mitochondrial dysfunction, operationally defined as a decreased respiratory capacity per mitochondrion together with a decreased mitochondrial membrane potential, typically accompanied by increased production of oxygen free radicals, is a cause and a consequence of cellular senescence and figures prominently in multiple feedback loops that induce and maintain the senescent phenotype. Here, we summarize pathways that cause mitochondrial dysfunction in senescence and aging and discuss the major consequences of mitochondrial dysfunction and how these consequences contribute to senescence and aging. We also highlight the potential of senescence-associated mitochondrial dysfunction as an antiaging and antisenescence intervention target, proposing the combination of multiple interventions converging onto mitochondrial dysfunction as novel, potent senolytics.

## Introduction

### What is cell senescence?

Cell senescence was originally defined as the irreversible loss of replicative potential of primary cells in culture (1), initiated as a persistent DNA damage response to dysfunctional telomeres (2). Telomere dysfunction might be caused by telomere shortening during DNA replication (3, 4), replication-independent telomere damage (5), or a variety of other stresses (6). Such stresses often, but not always, involve a persistent DNA damage response (7). Senescent cells accumulate with aging in many mammalian and non-mammalian tissues (8), and intervention experiments (9–11) have proven that this accumulation is a physiologically important driver of age-associated functional decline, (multi-)morbidity, and mortality (for review, see ref. 12). In addition to an activated DNA damage response, senescent cells are characterized by a multitude of “building blocks,” the interaction of which appears necessary and sufficient to stabilize and maintain the senescent phenotype (13). These include deregulated nutrient signaling (14, 15), autophagy/mitophagy dysfunction (16), and massive epigenetic reprogramming (17, 18), including activation of (proinflammatory) transcription factors driving the senescence-associated secretory phenotype (SASP) (19–22). This list is not exhaustive; for instance, apoptosis resistance might also be regarded as an essential building block of the senescent phenotype (11). In the present context, however, the most important of these building blocks is senescence-associated mitochondrial dysfunction (16). Markers for activation of the same building blocks were also found in aged postmitotic cells (23), indicating that a senescence-like phenotype is a general stress response for both proliferation-competent and differentiated postmitotic cells during aging (24). For more detailed overviews of the senescent phenotype, the reader is referred to recent reviews (6, 20, 25–27).

### What is mitochondrial dysfunction?

**Figure 1:**
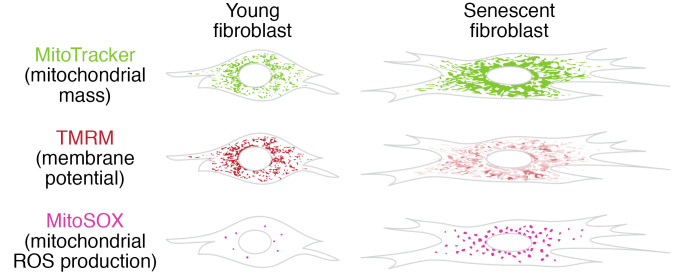
Mitochondrial dysfunction in senescence. Illustrations are representative of mitochondrial mass, membrane potential, and ROS levels in young versus senescent fibroblasts as observed after staining with fluorescent dyes. Mitochondria in human fibroblasts can be stained with MitoTracker (green) to show mitochondrial mass and tetramethylrhodamine methyl ester (TMRM) (red), which accumulates in mitochondria in a membrane potential–dependent fashion at under-saturated concentrations. There is higher mitochondrial mass in senescent fibroblasts, but their membrane potential is lower (as indicated by weak and patchy TMRM staining) than in non-senescent (young) fibroblasts. The mitochondrial network is more fragmented in young cells, while mitochondria are fused in senescence. Mitochondrial superoxide levels can be visualized using MitoSOX (pink). Mitochondrial superoxide levels are elevated in senescent human fibroblasts.

The defining feature of mitochondrial dysfunction in aging tissues and senescent cells is a decrease in respiratory capacity per mitochondrion together with a decreased mitochondrial membrane potential (MMP) at steady state. Mitochondrial mass is often increased in senescence in vitro (28–30) and in vivo (31). In vitro, this might partially be a consequence of increased total cell mass in senescence. However, because mitophagy is decreased in senescence (see below), dysfunctional mitochondria accumulate and the increased mitochondrial mass may partially compensate for loss of mitochondrial function (refs. 29, 30, and Figure 1). During mitochondrial dysfunction, low MMP is typically associated with increased production of reactive oxygen species (ROS) (32, 33); however, empirical determination of ROS is required for each case to confirm this. Transient reduction in MMP renders the electron transport chain (ETC) in a more oxidized state and can reduce ROS production, but sustained reduction in MMP may indicate ETC dysfunction. Alternatively, a low MMP might be due to increased proton leak, for example through upregulation of uncoupling proteins. As complex I in the ETC oxidizes the reduced form of nicotinamide adenine dinucleotide (NADH) to NAD^+^, decreased respiratory capacity may contribute to the reduced NAD^+^/NADH balance found in aging and in cell senescence. In contrast, in oncogene-induced senescence, the NAD^+^ salvage pathway was upregulated, leading to enhanced SASP production via AMPK suppression (34).

Defects in oxidative phosphorylation (OXPHOS) function have been observed in various models of senescence, confirming the causal relationship between senescence and OXPHOS inefficiency. For example, iron chelation–induced senescence involved decreased complex II activity, which preceded the activation of p27kip1-mediated cell cycle arrest in Chang normal liver cells (35). Similarly, TGF-β1–induced senescence of Mv1Lu mink lung epithelial cells involved the inhibition of complex IV, which caused mitochondrial ROS generation and the persistent disruption of MMP, triggering senescence (36). These findings emphasize that mitochondrial OXPHOS dysfunction is a common feature of senescent cells.

There are widely varying results concerning the importance of individual ETC complexes for loss of respiratory capacity. For instance, in heart muscle (especially in interfibrillar mitochondria), it is primarily complexes III and IV that lose activity with age, while in skeletal muscle, liver, and brain, complex I often appears more sensitive to age-associated loss of function (37, 38). The tissue and model examined as well as methodological differences between studies might account for these apparent differences. As the largest respiratory chain complex, complex I function and ROS production are dependent on assembly fidelity, which decreases with age, while knockdown of a single complex I assembly factor was sufficient to induce cell senescence (38). Respiratory chain complexes form supercomplexes. Their stability appears to decrease with aging (39), and it has been speculated that this may contribute to increased ROS production (40).

Thus, mitochondrial dysfunction and senescence are closely interconnected hallmarks of aging. In the following sections, we will first highlight the key mechanisms that contribute to mitochondrial dysfunction in aging and senescence. Then we will discuss the impact of mitochondrial dysfunction on the senescent phenotype.

### Assessing mitochondrial (dys-)function.

Functional parameters of mitochondria can change rapidly and massively, reflecting cellular needs. For example, under a high-fat diet, the abundance of fatty acid oxidation enzymes increases, while under oxidative stress, antioxidant enzymes are upregulated. Mitochondria in cultured cells under contact inhibition (confluent state) are more coupled than mitochondria in cells that are replicating (41). Exercise rapidly increases biogenesis of key mitochondrial enzymes in the ETC in skeletal muscle (42). Such changes are controlled via potent feedback loops to the nucleus (termed “retrograde response”) that aim to maintain mitochondrial ATP generation. Thus, mitochondrial functional changes could reflect either an appropriate response to a particular signal, or an inability to maintain mitochondrial activities appropriately (dysfunction), making cause-and-effect relations difficult to untangle.

The best determinant of mitochondrial function is the ability to maintain MMP in response to a particular stimulus, as this defines the ability of the mitochondria to generate ATP efficiently. The MMP depends on sequential intramitochondrial biochemical reactions that are linked to the oxidation of respiratory substrates in the Krebs cycle. Proton pumping through the ETC results in the generation of an electrochemical gradient between the intermembrane space and the mitochondrial matrix. The H^+^ gradient drives the synthesis of ATP from ADP and inorganic phosphate by the ATP synthase (complex V), coupling electron flow in the ETC to ATP synthesis (“coupled respiration”). It also assists the transport of ions and metabolites across the inner mitochondrial membrane as well as the import of mitochondrial proteins. The proton leak dissipates the electrochemical gradient, making mitochondrial ATP production less efficient.

Tightly coupled mitochondria are typically able to increase oxygen consumption rates rapidly several fold from the resting state (defined by the absence of external fuel substrates and exhaustion of endogenous substrate, called state 2 by Chance and Williams [ref. 43]) to the ADP-phosphorylating state (in which fuel substrate is available and which supports coupled energy transformation, termed state 3). Experimentally, the ATP synthase inhibitor oligomycin can generate a “resting state” that indicates the basal ETC activity driven by the proton leak in the absence of ATP synthase activity (state 4). Then, the ratio of state 3/state 4 respiration rates, referred to as the respiratory control ratio (RCR), indicates the degree of coupling of mitochondria and is a measure of the efficacy of ATP production and the ability to maintain the MMP. For a more detailed review of mitochondrial physiology, the reader is referred to an excellent recent review (44).

RCR is a powerful diagnostic tool to assess mitochondrial function, as almost any aspect of OXPHOS can influence it. However, there is no absolute and decisive RCR value that indicates mitochondrial dysfunction, as values are substrate and cell type dependent. While the RCR can be empirically determined from isolated mitochondria or permeabilized cells, it cannot be directly measured in intact cells, because respiratory states cannot be definitively controlled. Nonetheless, apparent respiratory control can still be determined in intact cells (for an excellent review, see ref. 45). For measurement of MMP in intact cells using potentiometric fluorescent dyes like tetramethylrhodamine methyl ester (TMRM), tetramethylrhodamine ethyl ester (TMRE), and rhodamine 123, careful experimental methodologies need to be employed (45). MMP indicator dyes such as JC-1 and DiOC_6_(3) (46) and indicator dyes for mitochondrial superoxide (MitoSOX, Thermo Fisher Scientific) (47) are subject to artifacts and should be used only with extreme caution.

## Senescence drives mitochondrial dysfunction

**Figure 2:**
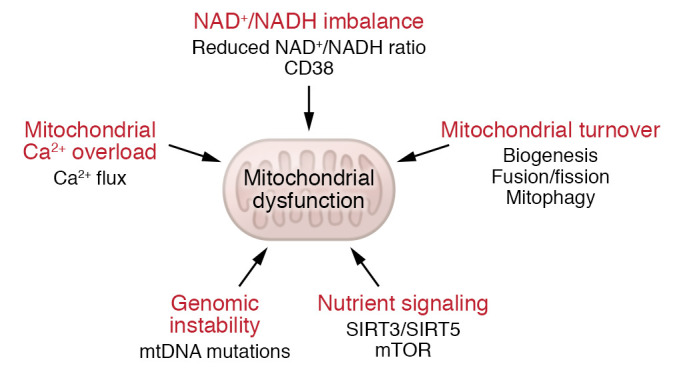
Mechanisms that can cause mitochondrial dysfunction. These include mtDNA mutations (genomic instability); mitochondrial turnover (as defined by the ratio of mitochondrial biogenesis and mitophagy, associated with fusion and fission); nutrient signaling through mTOR, modified by the mitochondrial sirtuins SIRT3 and SIRT5; the NAD^+^/NADH ratio (which is controlled by CD38, among others); and Ca^2+^ fluxes resulting in mitochondrial Ca^2+^ overload. See text for discussion.

The impairment of mitochondrial function, as well as its morphology, has been associated with aging and many aging-associated pathological conditions, including cancer, neurodegenerative diseases, metabolic diseases, kidney diseases, and others (33, 48, 49). Mitochondrial dysfunction and associated ROS production has also been found in stress-induced senescence (28, 32), replicative senescence (50), oncogene-induced senescence (7, 50), and senescence triggered by genetic telomere uncapping (32). Importantly, specific ablation of mitochondria from senescent cells was sufficient to reverse many features of the senescent phenotype (31). Mechanisms that contribute to mitochondrial dysfunction in aging and senescence are summarized in Figure 2 and are discussed below.

### Genomic instability.

Mitochondrial DNA (mtDNA) encodes 13 proteins crucial for electron transport as well as the genes for the 12S and 16S rRNAs and for 22 transfer RNAs. Damage to the mtDNA replication system and/or mtDNA repair mechanisms can severely affect mitochondrial function. mtDNA mutations or deletions causing mtDNA damage have been implicated in several mitochondrial diseases (51, 52). mtDNA mutations accumulate with age in postmitotic tissues (53) and expand clonally in stem cell compartments (54). The mechanistic link of mtDNA damage involved in aging was proven by studies involving defective proofreading activity of mtDNA polymerase. These studies showed that at high density, mtDNA mutations cause physiologically relevant mitochondrial dysfunction and premature aging (55, 56). mtDNA deletions were later identified as driving premature aging in these mice (57). While neither increased ROS nor cell senescence was observed in the original publications, this was later rectified (58), and a specific type of cell senescence missing the proinflammatory arm of the SASP was found in adipose tissue of PolG mutator mice, which rapidly accumulate mtDNA mutations (59) (see below). mtDNA depletion (i.e., ρ^0^ cells) can cause cellular senescence, specifically if associated with high ROS levels (60). In contrast, complete ablation of mitochondria rescues many features of senescence (31). A specific point mutation in the control region of mtDNA (T414G) that accumulates in aging skin was not associated with skin fibroblast senescence (61). Altogether, there is little evidence that mtDNA (point) mutations at levels that occur during healthy aging are sufficient to drive widespread mitochondrial dysfunction and senescence (33).

### Impairment of mechanisms involved in mitochondrial mass homeostasis.

Mitochondrial mass homeostasis involves various mechanisms, such as mitochondrial biogenesis, clearance of damaged mitochondria by mitochondria-specific autophagy (mitophagy), and maintenance of mitochondrial dynamics (62). Impairments in these processes may lead to mitochondrial dysfunction. The PPARγ coactivator 1 (PGC-1) family, comprising PGC-1α, PGC-1β, and PGC-1–related coactivator, are main regulators of mitochondrial biogenesis (63). Nuclear-encoded mitochondrial transcription factor A (TFAM) is another important factor that regulates mitochondrial biogenesis and plays a fundamental role in mtDNA transcription (64). PGC-1α activates the transcription factors Nrf1/2 and ERRα, which further control the TFAM transcription in response to increased mitochondrial biogenesis (65). PGC-1α is induced under physiological stresses like exercise and calorie restriction/fasting to increase mitochondrial biogenesis. It also affects mitochondrial function by regulating the activity of other transcription factors like PPARγ, YY-1, and GABPA. Several studies have revealed a role for PGC-1α in mitochondrial dysfunction associated with pathological states (66, 67).

In the context of senescence, mitochondria of senescent cells undergo structural changes that are typically associated with significant increases in size and volume. Increased mitochondrial mass and PGC-1α and Nrf1 and/or Nrf2 induction were associated with replicative (68) and oncogene-induced (69) senescence. However, the increase in mitochondrial mass results mainly from the accumulation of dysfunctional mitochondria due to alterations in the mitophagy degradation process (29, 70). Among different mitophagy pathways, PINK1/parkin is the most studied stress-induced mitophagy pathway (71), and its impairment has been associated with several pathological conditions. Recent studies revealed that excessive *S-*nitrosylation of parkin resulting in decreased fission was responsible for altered mitophagy in senescence (30). However, the role of autophagy in senescence has been conflicting. Some studies showed that increased autophagy favors senescence, is correlated with a negative feedback from the PI3K/mTOR pathway, and might be required for the transition to senescence (72). In contrast, other studies show that impairment in autophagy pathways can induce premature senescence, possibly due to generation of ROS by mitochondrial dysfunction, which in turn activates p53 signaling in primary human fibroblasts (73) and in muscle stem cells (74). Cigarette smoke has been shown to induce senescence in lung fibroblasts and small airway epithelial cells by inhibiting mitophagy (75). Induction of mitophagy via inhibition of the mTOR pathway postpones senescence (16, 29, 31, 76). Furthermore, inhibition of selective autophagy for the transcription factor GATA4 was associated with induction of senescence (77). We speculated that general autophagy and mitophagy might be distinctly regulated in senescence (16). Direct comparative studies of general autophagy, mitophagy, and selective autophagy during establishment and stabilization of senescence will be necessary to clarify this issue.

Changes in mitochondrion morphology through fission and fusion processes, known as mitochondrial dynamics, are crucial to maintain mitochondrial number, size, shape, and distribution. Mitochondrial fusion allows the exchange of damaged mtDNA with intact mtDNA and involves fusion proteins such as mitofusin 1 and 2 (Mfn1/2) and optic atrophy protein 1 (OPA1). Mitochondrial fission produces new mitochondria by recruitment of Drp1 protein to the surface of mitochondria by receptors like FIS1, Mff, and mitochondrial dynamics proteins such as MiD49 and MiD51. Interference with Mff-Drp1 interactions is associated with mitochondrial dysfunction (78).

Perturbations in mitochondrial dynamics have been associated with cellular senescence. Increased oxidative damage due to accumulated dysfunctional mitochondria reduces FIS1, leading to formation of giant mitochondria (79). Mitochondrial elongation has been associated with induction of senescence-associated pathways (79, 80).

### Dysregulated nutrient sensing pathways.

Nutrient sensing pathways identify fluctuations in nutrient supply and initiate cellular adaptations in response to energy demand. Several metabolic regulators of nutrient sensing mechanisms such as insulin/IGF-1, mTOR, AMPK, and sirtuins have been implicated in aging. Excessive cellular nutrients activate the insulin/IGF-1 and mTOR pathways, leading to the induction of anabolic processes and inhibition of autophagy (81). On the other hand, AMPK and sirtuins are activated in nutrient scarcity, like fasting or calorie restriction, leading to mitochondrial biogenesis and increased autophagy (82). Therefore, dysregulation of these nutrient sensing pathways can cause mitochondrial dysfunction. For example, higher expression of mTORC1 is associated with reduced mitophagy, resulting in accumulation of defective mitochondria (83). In senescence, mTOR is persistently activated (15), which may be a consequence of elevated ROS produced from dysfunctional mitochondria (84). Under low-energy conditions, metabolic reprograming of cells involves activation of AMPK (85). AMPK activation promotes mitochondrial biogenesis and regulates mitochondrial dynamics and mitophagy (86), partially by promoting SIRT1-dependent PGC-1α deacetylation by increasing intracellular NAD^+^/NADH ratio (87).

Sirtuins are NAD^+^-dependent deacetylases that also act as metabolic sensors and play important roles during stress and in cell metabolism. Sirtuins regulate mitochondrial biogenesis and modulate the composition and function of the mitochondrion. In mammals, three members of the SIRT family, SIRT3, SIRT4, and SIRT5, are localized in mitochondria and modulate mitochondrial metabolism (88). SIRT3 plays a unique role as the main mitochondrial deacetylase (89). Loss of mitochondrial SIRT function, specifically loss of SIRT3, has been linked to many age-related diseases, including insulin resistance, cancer, cardiovascular diseases, and neurodegeneration (90). Depletion of SIRT3, and to some extent SIRT5, induced a senescence response with distinct SASP factors (59). Furthermore, CD38-regulated NAD^+^ decline and mitochondrial dysfunction in aging are mediated, at least in part, through SIRT3 (91). In addition, SIRT4 and SIRT5 also regulate mitochondrial signaling and are involved in rewiring various metabolic pathways (92, 93). Therefore, declines in mitochondrial sirtuin activity, such as observed in stress and aging, may impair proper mitochondrial response and function.

### Imbalanced NAD^+^/NADH ratio.

Diminished NAD^+^ levels lead to cellular and mitochondrial decline during aging. Normal mitochondrial function requires maintenance of optimal NAD^+^/NADH ratios as well as mitochondrial NAD pool. The ratio between NAD^+^ and its reduced NADH counterpart is tightly related to several cellular reactions and mitochondrial metabolism. NAD^+^ acts as a cofactor in many oxoreduction pathways and as a substrate for several redox reactions. The production of ATP and maintenance of MMP both require NAD^+^ as a cofactor. NADH is generated from glycolysis and TCA cycle in the mitochondria, whereas NAD^+^ can be regenerated through reactions like the oxidation of NADH by complex I. Cellular NAD^+^ levels are dynamically regulated by a balance between its synthesis and degradation processes. NAD^+^ can be synthesized via multiple pathways: the de novo synthesis pathway, the NAD^+^ salvage pathway, and the Preiss-Handler pathway (94, 95). NAD^+^-consuming enzymes like poly(ADP-ribose) polymerases (PARPs), sirtuins, SARM1, CD38, and CD157 degrade NAD and regulate the overall cellular NAD pool. Mitochondrial NAD^+^ biosynthesis is also modulated in response to nutritional and environmental stimuli. Although the levels of NAD^+^ in mitochondria seem to stay stable during genotoxic stress, extended NAD^+^ depletion can lead to mitochondrial dysfunction. In fact, NAD^+^ depletion causes mitochondrial membrane depolarization and mitochondrial permeability transition, which are involved in PARP1-mediated cell death (96). Accumulation of damaged DNA during aging causes the activation of PARP1, which in turn activates the DNA damage response. Since PARP1 is a NAD^+^-dependent enzyme, DNA damage can result in depletion of the NAD pool in a PARP1-dependent manner. This then further limits the NAD^+^ availability for sirtuins, leading to mitochondrial dysfunction.

In addition, recent studies have reported a role for the NAD^+^-consuming enzyme CD38 in NAD^+^ decline during aging (91, 97). The expression, as well as activity, of CD38 is induced during chronological aging, and this increase in CD38^+^ cells is mediated in part by the SASP of senescent cells, suggesting a strong link between cellular senescence and NAD^+^ decline during aging (97–99). Therefore, disruption in the NAD^+^/NADH ratio and the NAD pool can affect mitochondrial dysfunction, which may be partly responsible for the induction of senescence and aging.

### Calcium overload.

Mitochondria actively participate as modulators, buffers, and sensors to maintain Ca^2+^ homeostasis (100). This homeostasis is regulated by protein channels localized in the inner and outer mitochondrial membranes (IMM and OMM), and by crosstalk with the endoplasmic reticulum (101). Mitochondrial Ca^2+^ influx occurs through voltage-dependent anion channels (VDACs) present in the OMM, and Ca^2+^ then enters the mitochondrial matrix through the mitochondrial calcium uniporter (MCU) located in the IMM. On the other hand, mitochondrial Ca^2+^ efflux involves HCX and NCLX channels present in the IMM. Additionally, intracellular Ca^2+^ buffering is governed by inositol 1,4,5-trisphosphate receptor (IP3R), Grp75, and VDAC interaction (102). An increase in cytosolic Ca^2+^ concentration leads to rapid uptake of Ca^2+^ by mitochondria to prevent Ca^2+^ overload in the cytosol but may cause mitochondrial Ca^2+^ overload (100), which results in increased ROS generation and mitochondrial dysfunction including reduced ATP production (103). The various mechanisms involved in Ca^2+^ overload–induced mitochondrial dysfunction include Ca^2+^-induced nitric oxide production; Ca^2+^-enhanced cytochrome *c* dissociation from the IMM; opening of the mitochondrial permeability transition pore (mPTP), which subsequently causes the release of cytochrome *c* and GSH- and NADPH-dependent antioxidative enzymes; the arachidonic acid pathway; and Ca^2+^/calmodulin-dependent protein kinase II (CaMKII) activation (104, 105).

Mitochondrial Ca^2+^ overload instigates the mitochondrial metabolism impairment during senescence and age-related diseases (106). During senescence, activation of IP3R leads to Ca^2+^ release from the endoplasmic reticulum and causes accumulation of Ca^2+^ through MCU channels, leading to mitochondrial Ca^2+^ overload (107). This Ca^2+^ overload in mitochondria causes a decrease in membrane potential, increased ROS generation, and senescence.

## Mitochondrial dysfunction governs the senescent phenotype

In addition to mitochondrial dysfunction, senescent cells are characterized by high levels of DNA and specifically telomere damage, a persistent DNA damage response, an activated SASP, shifts in the NAD^+^/NADH ratio, activation of innate immune responses, and activation of antiapoptotic mechanisms. As discussed below, mitochondrial dysfunction contributes to all these senescent phenotypes and to age-associated cell and tissue alteration via multiple pathways. As these pathways may result in different outcomes, mitochondrial dysfunction might be a major determinant of the variability of the senescent phenotype between different cell types, senescence inducers, and/or tissue contexts.

### ROS link mitochondrial dysfunction and telomere-dependent, proinflammatory senescence.

Impaired mitochondria with inefficient OXPHOS produce excessive ROS. This increase in oxidative stress leads to DNA damage, such as oxidized bases, single-strand breaks, double-strand breaks, and telomere shortening (4), activating p53 and pRb pathways and causing cell cycle arrest and senescence (28, 108). Furthermore, since mitochondria play a role in nucleotide synthesis, mitochondrial dysfunction may affect telomere attrition and genome stability via attrition of nucleotide pools (109).

Importantly, in telomere-dependent (including replicative and stress-dependent) senescence, telomere uncapping itself induced mitochondrial dysfunction in an ATM- and p21-dependent manner, creating a positive-feedback loop (32). In addition, telomere shortening can activate p53 signaling, which inhibits PGC-1α and PGC-1β promoters, resulting in enhanced mitochondrial dysfunction (110). The same type of mitochondrial dysfunction was also found in oncogene- (RAS-)induced senescence (69). This senescence-induced mitochondrial dysfunction was associated primarily with failure of complex I–dependent respiration, accumulation of dysfunctional mitochondria, and enhanced ROS production. It was established early during development of the senescent phenotype, specifically before a proinflammatory SASP developed (32, 69). In fact, suppression of senescence-associated ROS blocked activation of NF-κB and inhibited release of IL-6 and IL-8 from senescent fibroblasts, while blocking NF-κB had no effect on ROS levels (50). Moreover, completely ablating mitochondria from senescent cells by parkin-mediated autophagy reduced multiple markers of senescence, including many components of the proinflammatory SASP (31). Together, these data identify mitochondrial dysfunction as the essential prerequisite for the formation of the full senescent phenotype, including a proinflammatory SASP, following telomere uncapping.

### Mitochondrial redox regulation, the NAD^+^/NADH ratio, and senescence.

Mitochondria are not only dependent on the NAD^+^/NADH balance for their proper function (see above); they are also major determinants of the cellular redox status and specifically the NAD^+^/NADH ratio. Mitochondria oxidize NADH generated by the TCA cycle or fatty acid oxidation within mitochondria and from the cytosolic NAD^+^/NADH pool via the α-glycerophosphate and malate-aspartate shuttles (111). Dysfunctional mitochondria are less effective, and in fact, reduced ratios of cytosolic NAD^+^/NADH were found in replicative senescence (112) and in aging (113). However, this might be primarily an indirect consequence of mitochondrial dysfunction. NAD^+^ and NADH levels are highly compartmentalized between mitochondria and cytoplasm, with a change in one compartment often having little impact on the other (114). However, mitochondrial dysfunction induces greater dependency on glycolysis, which enhances NAD consumption in the cytoplasm.

Cells with reduced NAD^+^/NADH ratio display elevated ADP/ATP and AMP/ATP ratios, which in turn activate AMPK, which can induce senescence by phosphorylating p53 and/or stabilizing p16INK4 mRNA (59). In fact, downregulation of the NAD^+^-dependent malic enzymes 1 and 2 (ME1 and ME2) triggered a p53-dependent senescence response, whereas overexpression of these enzymes suppressed senescence (115). The decline in the expression and activity of nicotinamide phosphoribosyltransferase (NAMPT), the rate-limiting enzyme in the NAD^+^ salvage pathway, led to replicative senescence, whereas its overexpression caused a delay in senescence induction mediated by increased activity of SIRT1 (112, 115). Induction of mitochondrial dysfunction by depletion of either SIRT3 or SIRT5, depletion of mtDNA by ethidium bromide, or treatment with the ETC inhibitor rotenone or antimycin A all reduced the cytosolic NAD^+^/NADH ratio and induced a senescent growth arrest. Similarly, tissues from PolG^D257A^ mice, which accumulate massive mtDNA mutations (55, 56), also showed widespread senescence associated with low NAD^+^/NADH ratios. Interestingly, in all these cases, the resulting senescence was characterized by a SASP that largely lacked its proinflammatory arm, including NF-κB activation and hyperproduction of IL-1, IL-6, and IL-8. Rather, the SASP of these cells was characterized by high levels of IL-10, CCL27, and TNF-α (59).

The physiological relevance of this type of senescence induced by severe interference with mitochondrial function remains to be established. However, it might be of interest to compare it with Notch-controlled senescence. During the early phase of oncogene- or stress-induced senescence, i.e., before induction of a proinflammatory SASP, cells produce a TGF-β– and growth factor–rich secretome under the control of Notch1, which only switches over to the proinflammatory secretome when Notch1 activity ceases after a few days (116). Whether mitochondrial dysfunction plays a role in Notch-dependent senescence, and what this role could be, are unclear. However, reaction kinetics suggests this as a possibility: in stress-induced senescence, mitophagy is suppressed already within a few hours following stress (70), i.e., at the same time when the Notch1-dependent SASP is being generated.

It is not known whether and to what extent senescence induced by severe mitochondrial dysfunction occurs under conditions of physiological aging. In oncogenic RAS-induced senescence, HMGA proteins upregulated the expression of NAMPT, causing an *increase* in the NAD^+^/NADH ratio leading to enhanced glycolysis, suppression of AMPK, and suppression of the p53-mediated inhibition of p38 MAPK. This enhanced NF-κB activity and induced the “conventional,” proinflammatory SASP (34).

### Dysfunctional mitochondria contribute to innate immune response activation.

Dysfunctional mitochondria release different forms of danger-associated molecular patterns (DAMPs) that are recognized by the innate immune system and promote inflammation. For example, cardiolipin, a glycerophospholipid located in the IMM, can promote inflammation when it becomes exposed in damaged mitochondria (117). In senescent fibroblasts, cardiolipin accumulated and exposure of fibroblasts to cardiolipin was sufficient to induce senescence (118). Other mitochondrial DAMPs are ROS and mtDNA. Cell-free mtDNA increases during human aging, correlating with markers of sterile inflammation (119). mtDNA has the capacity to activate multiple immune response pathways, including the NLRP3 inflammasome (120, 121) and the cyclic GMP-AMP synthase (cGAS)/stimulator of interferon genes (STING) pathway (122). These innate immune responses are activated not only in aging (121, 123, 124), but also in cell senescence. The NLRP3 inflammasome was activated via a ROS/thioredoxin-interacting protein pathway in senescent vascular epithelial cells (125) and contributed to senescence maintenance via an NLRP3/IL-1β positive-feedback loop (126). cGAS/STING identifies fragments of nuclear DNA referred to as cytoplasmic chromatin fragments (CCFs). This recognition of CCFs by cGAS leads to the activation of NF-κB signaling and drives the SASP in senescent cells (127, 128). Senescence in ATM-deficient cells was mediated by STING activation and could be prevented by boosting of NAD^+^ levels with nicotinamide riboside, which promoted mitophagy (129). Whether release of mtDNA from dysfunctional mitochondria is sufficient to activate cGAS/STING in senescence is not clear. However, a recent study showed that mitochondrial dysfunction associated with impaired nuclear-encoded mitochondrial OXPHOS genes activates the ROS/JNK retrograde response that drives CCF formation and thus SASP (130). Thus, the mitochondria-to-nucleus retrograde signaling pathway is responsible for CCF formation during senescence.

### Mitochondrial membrane permeabilization and apoptosis resistance.

The mPTP, a highly regulated megachannel, is both an important underlying mechanism of, and an output pathway for, mitochondrial dysfunction in aging and, potentially, senescence. Oxidative stress, Ca^2+^ overload, IMM depolarization, and other stresses induce the (pathological) opening of the mPTP. Full opening of the mPTP causes membrane potential loss, increased production of mitochondrial ROS, and reduction of Ca^2+^ buffering capacity, thus forming a vicious cycle (131). However, short and/or infrequent opening of the mPTP can induce protective pathways, at least partially via “mitohormesis,” a term describing the response to small amounts of mitochondrial stress that can have beneficial effects on organismal health (132). During aging, mPTP activation becomes enhanced and may regulate ROS production in an adaptive (hormetic) or maladaptive manner (133, 134). It is not clear yet whether mPTP opening contributes to MMP decrease and ROS production in cell senescence.

The molecular makeup of the mPTP is still under debate, but it seems to combine subunits located in both the IMM (ANT, F_0_F_1_ATPase, CypD) and the OMM (VDAC, TSPO) (135). This indicates that the mPTP can translate IMM depolarization into a limited OMM permeabilization. Indeed, release of cytochrome *c* and other apoptotic factors, sufficient to trigger cell death in the extreme case, is one outcome of prolonged mPTP opening (131, 135). Moreover, the regulation of mPTP opening is dependent on proapoptotic BCL-2 family proteins BAX and BAK resident in the OMM (136).

In this context, it is interesting to note that OMM permeabilization in a relatively small number of mitochondria (termed “minority MOMP”) can be induced by mild, sublethal stress. This led to limited caspase activation that was insufficient to induce cell death but resulted in nuclear DNA damage (137). In fact, in the cytoplasm of senescent cells mitochondria-derived peptides were found to be elevated and promoted the SASP (138). Following irradiation stress, release of mitochondrial endonuclease G had also been observed, which caused nuclear DNA damage and a senescence-like phenotype missing the “classical” proinflammatory SASP (139). Furthermore, multiple antiapoptotic pathways are upregulated in senescence (11, 140, 141), presumably in response to partial mitochondrial membrane permeabilization.

## Senescence-associated mitochondrial dysfunction as intervention target

Finding interventions that improve mitochondrial function in senescence in a highly specific way has been difficult, partly because of the complexity and variability of the mitochondrial dysfunction phenotype. However, treatments that more or less indirectly improved mitochondrial function have been successfully applied to reduce the SASP and to increase healthy lifespan. Although not easily translatable for broad human use, dietary restriction is still the gold standard for such interventions. Several lines of evidence showed that dietary restriction improved mitochondrial function and reduced ROS in comparison with age-matched controls in different tissues and species (142). This improvement of mitochondrial function was correlated with reduced SASP and senescent cell load (143) and postponed onset of age-related disease and disability (144). The dietary restriction mimetics rapamycin and metformin had similar beneficial effects. However, these interventions are indirect and multispecific, and it remains unclear to what extent their healthspan-promoting effects are due to their impact on mitochondrial function. For instance, metformin was shown to decrease cellular respiration and ROS production by inhibition of mitochondrial ETC complex I (145). However, the concentrations needed for complex I inhibition are considerably higher than those achievable by therapeutic intervention in vivo. It appears that metformin reduces ROS and SASP in senescent cells in vivo mainly by suppressing the activity of cytoplasmic NADPH oxidases, specifically NOX4 (70).

On the other hand, we would like to speculate that the dysfunctional nature of senescent cell mitochondria could be an advantage for interventions that aim to induce senescent cell apoptosis.

Anti-senescence interventions, including both senolytic approaches (which aim to specifically ablate senescent cells) and senostatic/senomorphic approaches (designed to block the SASP and thus the proliferation of senescence via bystander signaling), have been extraordinarily successful in relieving a very wide range of broadly age-associated degenerative conditions in experimental mice, and clinical trials for many of these are ongoing (for review, see refs. 12, 146). BH3 mimetics, prominent among them ABT-263 (navitoclax), have been validated as senolytic drugs for certain types of senescent cells, based on increased expression of the antiapoptotic BCL-2 family members BCL-xL, BCL-2, and BCL-w in senescent cells (140). By inhibiting antiapoptotic BCL-2 family proteins, drugs like ABT-263 enable the proapoptotic BCL-2 family members to permeabilize the mitochondrial membrane, leading to release of cytochrome *c*, caspase activation, and apoptosis. Moreover, inhibition of antiapoptotic BCL-2 family members causes prolonged mPTP opening, which, in contrast to apoptosis induction, does not require BAX and BAK activation by oligomerization (136). However, similar to its limitations as an anticancer drug, ABT-263 has relatively low specificity and sensitivity for senescent cells. As a senolytic, it is typically used in concentrations at which toxicity to non-senescent cells becomes an issue. This is why the low MMP of senescent cells might be advantageous to induce synthetic lethality.

Like many cancer cells, senescent cells have less capacity to maintain MMP compared with normal cells and are thus exposed to prolonged mPTP opening and, possibly, to minority MOMP, suggesting MMP as a selective functional target for senescent cells. We expect that low doses of mitochondrial uncouplers, such as FCCP or CCCP, will lead to a persistent depolarization of the mitochondrial membrane in senescent cells, resulting in constant mPTP opening and cell death. However, the same doses of uncoupler should be well tolerated by non-senescent cells with their more robust OXPHOS machinery and thus better ability to maintain MMP. Accordingly, combination of a senolytic drug, specifically a BH3 mimetic, with an uncoupler should enhance senolytic sensitivity and specificity, enabling therapeutic efficacy to be reached at substantially lower doses of senolytic drugs, thus broadening the therapeutic window and reducing the risk of side effects for senolytic interventions. Experiments to test this proposition are being performed presently in our laboratory and by others. Further research into the nature of mitochondrial dysfunction in senescent cells will no doubt instigate more new ideas for therapeutic targeting of senescent cells, either toward their destruction or maybe even toward functional improvement.

## Conclusions

Mitochondrial dysfunction is an essential part of the senescent phenotype. It is linked with all other building blocks of senescence via multiple feedback loops. While there has been considerable progress in recent years, there are still important open questions, including: (a) What is the most important driver of mitochondrial dysfunction during induction of senescence? (b) Why do mitochondrial fission and mitophagy decrease in senescent cells? (c) Does low MMP resemble a targetable vulnerability of senescent cells? (d) Can mitochondrial dysfunction explain the variability of the senescent phenotype? (e) Is it possible to restore mitochondrial function in senescence and aging? Finding answers to some of these questions may well help to identify interventions to improve healthy aging.

## Version 1 07/01/2022

Electronic publication
